# System Performance and Process Capability in Additive Manufacturing: Quality Control for Polymer Jetting

**DOI:** 10.3390/polym12061292

**Published:** 2020-06-04

**Authors:** Razvan Udroiu, Ion Cristian Braga

**Affiliations:** Department of Manufacturing Engineering, Transilvania University of Brasov, 29 Eroilor Boulevard, 500036 Brasov, Romania; braga.ion.cristian@unitbv.ro

**Keywords:** additive manufacturing, material jetting, polymer, machine capability, process capability, statistical process control, quality, variability, tolerance grade

## Abstract

Polymer-based additive manufacturing (AM) gathers a great deal of interest with regard to standardization and implementation in mass production. A new methodology for the system and process capabilities analysis in additive manufacturing, using statistical quality tools for production management, is proposed. A large sample of small specimens of circular shape was manufactured of photopolymer resins using polymer jetting (PolyJet) technology. Two critical geometrical features of the specimen were investigated. The variability of the measurement system was determined by Gage repeatability and reproducibility (Gage R&R) methodology. Machine and process capabilities were performed in relation to the defined tolerance limits and the results were analyzed based on the requirements from the statistical process control. The results showed that the EDEN 350 system capability and PolyJet process capability enables obtaining capability indices over 1.67 within the capable tolerance interval of 0.22 mm. Furthermore, PolyJet technology depositing thin layers of resins droplets of 0.016 mm allows for manufacturing in a short time of a high volume of parts for mass production with a tolerance matching the ISO 286 IT9 grade for radial dimension and IT10 grade for linear dimensions on the Z-axis, respectively. Using microscopy analysis some results were explained and validated from the capability study.

## 1. Introduction

The applications of additive manufacturing (AM) to industry have developed from rapid prototyping (RP) and rapid tooling to rapid manufacturing (RM). Additive manufacturing will revolutionize future manufacturing as a key technology in the implementation of the new industrial revolution, Industry 4.0 [1].

Nowadays, the AM processes defined by ISO/ASTM 52900-15 [2] standard are starting to find applications in industry. An industrial additive manufacturing system [3] should have six main components: design, pre-processing, manufacture, post-processing, quality control, and maintenance. The performance of AM systems is an important task to be estimated for the production of parts in an industrial process. There are many AM processes [2] and technologies associated with them, as follows:

- Vat photo-polymerization (VP) process with the stereolithography (SLA) technology;
- Binder jetting (BJ) process with 3D inkjet printing (3DP) technology;
- Material extrusion (ME) process with the fused deposition modeling (FDM) technology;
- Material jetting (MJ) process with polymer jetting (PolyJet) and multi-jet printing (MJM) technologies;
- Sheet lamination (SL) process with the laminated object manufacturing (LOM) technology;
- Powder bed fusion (PBF) process with selective laser sintering/melting (SLS/SLM) and electron beam melting (EBM) technologies; and
- Directed energy deposition (DED) process with laser engineered net shaping (LENS) technology.

Polymers have become very popular as materials for AM, being used in most of the AM processes and targeting a variety of applications [4]. The performance of all the AM systems that are connected to the mentioned AM processes should be analyzed in order to determine their capability to produce parts for the industry. The artifacts or test pieces are primarily used to quantitatively assess the geometric performance of AM systems [5]. Additionally, the AM product characterization needs other tests such as feedstock materials characterization, mechanical tests [6,7], and surface texture characterization [8,9,10]. The test artifacts are intended to reveal the strengths and weaknesses of different additive manufacturing techniques. Furthermore, they allow the comparison of the performances of different AM systems and the same AM system over time [11]. According to [5], three main characteristics, accuracy, resolution, and surface texture, of the AM systems can be estimated based on some standardized artifacts. Thus, seven artifact geometries have been proposed as follows: linear and circular artifact artifacts to test the accuracy, pins, holes, ribs, and slots artifacts to test the resolution, and the surface texture artifact to test the texture of the surfaces.

Current geometric dimensioning and tolerancing (GD&T) standards have been developed based on the capabilities of traditional manufacturing processes as subtractive manufacturing and formative manufacturing methodologies [12]. New GD&T standards need to be implemented for the different AM processes that use a large variety of materials (plastics, metals, composites, ceramics etc.). The effect of process parameters on the mechanical and geometric performances of polylactic acid (PLA) based composite materials was investigated in [13,14,15] and the results show the great potential of 3D printed composites in different applications. The physical and chemical properties of polymers relevant to dimensional accuracy require different evaluation and quantification of geometrical tolerances in comparison to metal materials. The tolerance standards applicable for metal parts, therefore, cannot be adopted for plastic structures or can only be applied to a very limited extent.

In the production process, the variations and fluctuations in the manufacturing accuracy are influenced by many factors such as machines, workpiece, methods, people, and environment, etc. The inherent fluctuations have less impact on product quality [16]. The abnormal variations have a large impact on product quality [17]. The most known methods used to control and reduce the manufacturing process variation are the statistical process control, measurement system analysis, six sigma method, and Taguchi’s design of experiments [18]. 

Statistical process control (SPC) uses statistical methods in quality control to monitor, maintain, and improve the capability of manufacturing processes to assure product conformance [16,19]. Akande et al. [20] analyzed quality characteristics of strength, bending stiffness, density, and dimensional accuracy of parts built by the SLS process using SPC control charts. They concluded that SPC ensures consistency in product quality for long term production.

Any quality control process needs to quantify, first, the machine capability (short-term study or machine performance) in one continuous production run and manufacturing process capability (long-term study) in series production [19,21]. Measurement process capability provides the evidence for conformity or nonconformity with specification according to ISO 14253:2017 [22]. 

Experimental and theoretical studies have been developed in order to characterize the performance of AM processes and have particularly focused on quality control in additive manufacturing. Additionally, the standards focused on AM systems are under development. The use of AM processes in mass production depends on the part quality. Some issues are the inconsistency of AM repeatability and reproducibility that have not been solved yet for all the AM processes. Singh et al. [18] analyzed the repeatability of acrylonitrile butadiene styrene (ABS) replicas built by the FDM process and chemical vapor smoothing, but the repeatability variation and the appraiser variation were not calculated. Baturynska [23], using statistical analysis, attempted to improve the dimensional accuracy of the parts built by polymer powder bed fusion. She developed linear regression models to predict the value of the thickness, width, and length of rectangular specimens and to compensate for the shrinkage effect. The material jetting process allows time between the jetting of each layer of material to relieve internal stresses [24]. George et al. [24] reviewed the accuracy and reproducibility of 3D printed medical models from polymers, using material extrusion (FDM), powder bed fusion (SLS), binder jetting, and material jetting. They concluded that regular testing of the accuracy of AM systems and preventive maintenance are necessary steps for quality assurance. Preißler et al. [25] investigated a process capability for a fused filament fabrication (FFF) process using PLA material, based on a customized pyramid object manufactured in 25 samples. The results for a 30 mm dimension and tolerance of ±0.2 mm through the quality control chart shows that the process was not in the statistical control.

Singh [26] investigated the process capability of the linear dimensions of a prismatic component built by PolyJet technology from an EDEN 260 machine. The results of this study suggested that that process lies in the ±4.5 sigma limit with regard to the dimensional accuracy of the chosen specimen. However, the variability of the measurement system was not performed and the number of 16 parts used to determine the process capability was too low, according to the capability standards [27]. Kitsakis et al. [28] investigated the IT (International Tolerance) grades for the dimensions of eight samples printed with deposition layers of 30 microns on the Objet Eden 250 3D printer, and they assigned the IT11 grade for it. The variability of the measurement system used in this study was not accomplished. Yap et al. [29] investigated the design capability and manufacturing accuracy of the PolyJet 3D printing process on an Objet500 Connex3 PolyJet printer using artifacts with customized features and concluded that the accuracy of the parts printed in glossy mode was better than that in matte finishing, but the minimum clearance gap for parts was obtained in a matte finish. Minetola et al. [30] evaluated the dimensional accuracy of three AM systems for polymeric materials using the ISO IT grades of an artifact from the GrabCAD library, building two replicas. They concluded that a smaller layer thickness provided higher dimensional accuracy of the part dimensions.

From the literature survey, the results are as follows:

- The AM artifacts are intended to investigate the strengths and weaknesses of additive manufacturing processes and they allow the comparison of the performances of different AM systems.
- The AM process control has an important role on the part quality, but there is a lack of adequate AM control methods and standards. There is still no AM standard for machine performance and process capability determination in mass production.
- Only a few research studies have focused on the repeatability, ISO IT grades, and process capability of polymer based AM systems.

Having benefits in terms of cost reduction and shorten of the time-to-market in products, the implementation of polymer-based AM technologies within production depends on the process capability and control.

The main aim of this article was to define a methodology for statistically analyzing the AM system performances and AM process control. A case study regarding the EDEN 350 AM system and polymer jetting process was conducted to validate the proposed basic methodology. 

## 2. Materials and Methods

### 2.1. New Methodology for Statistical Quality Tools in AM Production

The main objectives of the new methodology in AM are to define statistical quality tools based on standards for the assessment of the variability of the measurement system, the additive manufacturing repeatability, AM system capability or AM system performance, and AM process capability. This methodology includes experiments, statistical analysis, and results interpretation. SPC tools are used to provide the mean of identifying possible changes in the process [16].

**Figure 1:**
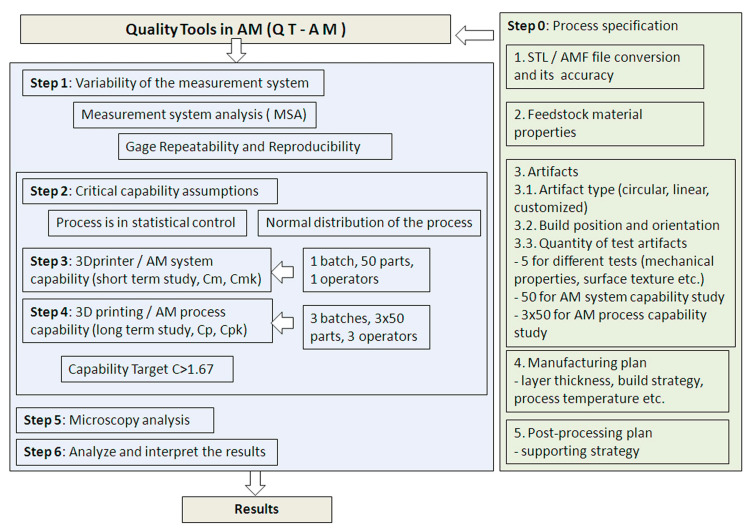
Flowchart of the proposed methodology/procedure named Quality Tools in AM (QT-AM).

The new methodology in AM consists of a preparatory step, followed by six main steps, as shown in Figure 1. 

The preparatory step defines the AM process specification as follows: STL (Standard Triangulation Language) or AMF (Additive manufacturing file) file conversion and its accuracy, feedstock material properties, artifact type, build orientation and position, the sample size of specimens, and the manufacturing and the post-processing plan. According to the ISO/ASTM 52901:2017 standard [31], the part definition made by AM, for a purchase purpose, should include the following characteristics: part geometry, tolerances, surface texture, feedstock material, build orientation, acceptable imperfections or deviations, and process control information (e.g., repeatability). The main characteristics of part geometry can be defined as a digital file containing the 3D model and a part engineering drawing. 

In traditional manufacturing, the specific requirements (dimensions, tolerances, surface finish, material, etc.) of the 3D model and drawings are set based on standards according to product material. Thus, ISO 286 is usually used for parts made of metal [32] and DIN 16742 for plastic parts [33]. In AM, the general tolerances for linear dimensions are specified according to the general standard ISO 2768-1 [34], based on the ISO/ASTM 52901:2017 recommendation. The surface texture or surface finish of the part should be specified by a maximum value. 

Feedstock material properties need to conform to the suppliers’ specifications. Artifact manufacturing should be undertaken according to a manufacturing plan (layer thickness, build strategy, process temperature). The CAD model of the artifact is converted to a STL file format. The conversion parameters used within different CAD software as well as any maximum deviation (chord height and angular tolerance) should be chosen correlated to the 3D printing layer thickness. Where supports cannot be avoided, a supporting strategy should be documented. It includes the support geometry, support material, the removal technique, and the specific post-processing treatments. The support material can be made from the same material as the artifact (model) material or can be different. The application of support structures or support material should be minimized on the critical features of the part.

The amount of variability induced in measurements by the measurement system itself should be determined before any capability study is performed (Figure 1). The measurements were performed using a grade “A” measurement method according to the ASTM 52902-19 standard [5]. Therefore, for simple and inexpensive measurements commonly available in a shop floor, a digital caliper was used. 

In the second phase of the methodology, the critical capability assumptions are analyzed, as shown in Figure 1. AM machine capability or AM system performance has the main purpose of the checking of existing 3D printers, objective arguments in case of 3D printer defects, and findings for target specifications when purchasing a new 3D printer. 3D printer/AM system capability and 3D printing/AM process capability studies are determined within the third and fourth steps. Capability is the ability of a system, or process, to realize a product that will fulfill the requirements for that product. Capability conditions under which the process is evaluated include the following, according to the ISO 22514-1:2014 standard [35]:

- Methods applied to demonstrate that the process is in control;
- Technical conditions (input batches, operators, tools, etc.);
- Measurement process (resolution, repeatability, reproducibility, etc.); and
- Data collection (duration, frequency).

Capability analysis should be carried out for a new or changed production process and then over time to control the process according to the standard ISO/TS 16949 [36]. Capability analysis is summarized in indices that show the system’s ability to meet its requirements. Machine and process capabilities provide results on how well a machine and a process performs in relation to defined tolerance limits. These two branches differ because they are determined in different conditions, but principally similar indices are calculated. The target capability indices commonly used in the automotive industry are greater than 1.67, which corresponds to a safety or critical parameter for a new process [16]. The quality condition is excellent if the capability indices are between 1.67 and 2 [16,37]. 

A quality inspection through a microscopy study is performed in the fifth step of the methodology. Optical micrographs were performed using a Zeiss O-Inspect (Carl Zeiss Industrielle Messtechnik, Oberkochen, Germany) multi-sensor measuring machines.

### 2.2. Process Specifications. Materials, Artifact, and Manufacturing Method

**Figure 2:**
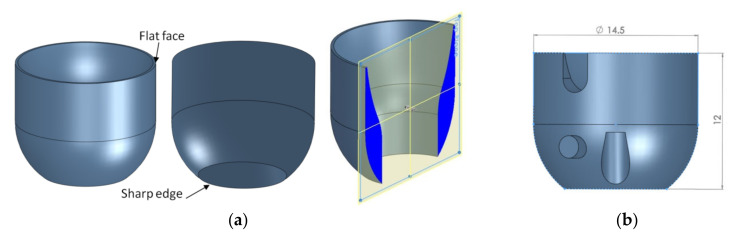
(**a**) Views and section view of the artifact. (**b**) The part used in the pre-production.

In this work, a part used in the pre-production of plastic parts has been selected as the benchmark. The part presents similar geometric basic features as the circular artifact shown in Figure 2. The circular artifact consists of a circular upper surface and a steep lower surface ending with a sharp edge (Figure 2). Two critical dimensions in terms of assembly and functionality of the artifact, the height H = 12 mm, and the diameter D = 14.5 mm, have been selected for the machine and process capability study. A fine tolerance class of ±0.1 mm was selected, taking into account the ranges of nominal lengths between 6–30 mm according to the ISO 2768-1 standard [34].

SolidWorks version 2013 software (Dassault Systèmes, Massachusetts, MA, USA) was used to design the 3D model and to generate the STL file. The 3D model of the part was converted into a STL file, which is the input file format of the Objet EDEN 350 PolyJet machine (Stratasys, Rehovot, Israel) [38]. The STL file conversion tolerances were set to a deviation of 0.01 mm and an angular tolerance of 4 degrees.

Feedstock materials used in this study were Objet VeroBlue RGD840 resin used as the model material and FullCure 705 as the support material [39]. The composition of the Objet VeroBlue RGD840 resin consists of an acrylic monomer, urethane acrylate oligomer, epoxy acrylate, and photo-initiator. FullCure 705 resin is made of an acrylic monomer, polyethylene glycol 400, propane-1, 2-diol, glycerol, and photo-initiator. The main properties of the Objet VeroBlue RGD840 material are shown in Table 1 [39]. Characteristics may vary if different orientations of specimens and test conditions are applied [6,7].

**Figure 3:**
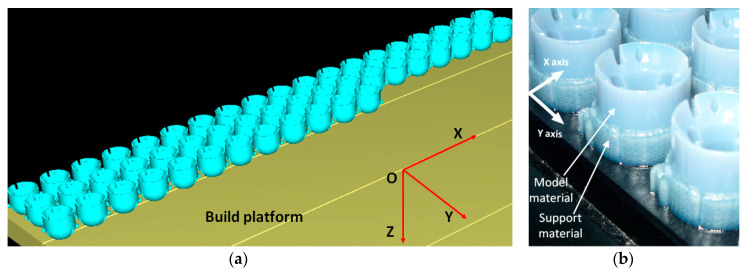
(**a**) Layout of the EDEN 350 build platform illustrating the 50 parts patterned in an array. (**b**) Detail of the printed specimens on the build platform.

The orientation of the specimens on the build tray affects how quickly, efficiently, and qualitatively they will be manufactured by the AM system [40]. Additionally, within the PolyJet process, the orientation of parts has an influence on the quantity and where the support material is used. The circular specimens were printed in a standing up position on the build platform, as shown in Figure 3. It is advantageous to print a circular model that has holes standing up on the build platform, so support material does not fill the holes [38]. Additionally, if a circular model is lying down on the build platform and printed in glossy printing mode, then the surface quality is affected by some errors [41]. The experimental roughness (Ra) values for the PolyJet material jetting process are specified according to the finish type as follows: for matte finish in the range of 0.5–15 μm, and for the glossy finish in the range of 0.5–4 μm [42]. The dimensional accuracy and the quality of the surface of a circular artifact built in standing up position are not significantly influenced by the orientation and positioning on the build platform. 

An Objet EDEN 350 PolyJet system was used to manufacture the specimens. Based on drop-on-demand (DOD) inkjet technology [43], the PolyJet system deposits layers of resin droplets of 0.016 mm thick. It levels each deposited resin layer and hardens it using ultraviolet (UV) light. During the process, the print heads and the photopolymer resins are heated at around 72 °C. The print heads were vacuumed at 6.2 atm. The experiments were performed under a controlled laboratory temperature of 20 °C and relative humidity of 30%. PolyJet 3D printers only use a solid infill pattern on parts. A different infill type can be added in the design stage of the CAD model, but the part’s interior will likely be filled with support material in the printing process. A solid infill pattern was used for all of the samples.

The build platform preparation (Figure 3), STL model slicing, and G-code generation were performed using the Objet Studio client/server software (Objet Geometries, Rehovot, Israel). The specimens were 3D printed in a glossy finish style. Only the bottom surfaces of the specimen were affected by the support material. The support material was removed with a pressure water jet from the bottom surface of the 3D printed specimens. 

The density of the printed material was determined using the Archimedes density method [44,45] by calculating the volume of five specimens, in addition to determining the mass of the parts using a precision scale. The results showed a mean density measured of the printed material of 1.15 g/cm^3^.

One batch of 50 parts was 3D printed for the AM system capability study, and three batches each containing 50 parts for the AM process capability study. A batch of 50 artifacts was manufactured in 1 h and 40 min, using 78 g of model material and 54 g of support material.

### 2.3. The Variability of the Measurement System

Within both manufacturing processes and quality systems, there is variation. All measurement data had some degree of variance or errors. A robust statistical process control (SPC) process requires accurate data to have the greatest impact on product quality. The percentage of variance due to the measurement system has to be determined. The measuring system can be affected by various sources of variation, called factors [46]: measuring instruments, operators, measuring method, specifications (the engineering tolerance), and parts or specimens. 

The variability of the measurement system was determined by Gage repeatability and reproducibility methodology. Repeatability is due to measuring instrument variation and reproducibility is due to operator (appraiser) variation. Gage R&R study was performed using the analysis of variance (ANOVA) method [47]. The ANOVA Gage R&R method estimates:

- The amount of measurement system variation compared with the process variation;
- The amount of variation in the measurement system that is due to operator influence; and
- The measurement system’s capability to discriminate between different parts.

The measurement system used in the analysis included:

- A Mitutoyo 500-196-30 digital scale caliper with advanced onsite sensor (AOS), a measuring range from 0 to 150 mm, and resolution 0.001 mm was used;
- The method that describes the way to keep the part in hand and the area to be measured for the height and for the diameter;
- A sample of 10 parts was used to be measured by three operators, twice, for each characteristic, the height, and the diameter. The parts were measured randomly;
- Circular 3D printed parts were made of polymers; and
- A controlled laboratory temperature of 20 °C and relative humidity of 30%.

Using Minitab 19 software (Minitab, Ltd., Coventry, United Kingdom) [48], a worksheet for Gage R&R analysis was created and the order of the measurements for each operator was imposed. The total sample size was 60 measurements.

### 2.4. System and Process Capability for PolyJet Technology

The Gauge R&R should be proven before the capability analysis. Two critical assumptions need to be considered when performing the machine and process capability analyses with continuous data, namely, the process is in statistical control, and a normal distribution of the process is required. A process is considered stable if its output is within the predictable limits. In order to assess whether or not a process is in statistical control, it uses control charts [16,19]. 

Short-term performance studies are typically performed on machines where parts are produced consecutively under repeatability conditions and the sample size produced is at least 50 workpieces to be manufactured in one shift [27]. 3D printer capability or AM system capability is used to assess the quality and performance of a single AM machine. The AM system capability was evaluated within the following conditions:

- 50 parts are printed at once;
- One operator manages the 3D printing process;
- The variation of the material batch or the printer user variation is not included in the total variation of the process; and
- The parts are measured and the data statistically analyzed.

The quality of the production processes is measured by establishing some characteristics and monitoring the long-term capability of its 3D printing process capability or AM process capability is a long-term study on a stable process that indicates the performance quality of the 3D printing process. The AM process capability can be evaluated within the following conditions:

- The specimens are 3D printed in three batches, each batch containing 50 specimens;
- Different operators manage the 3D printing process of the three batches based on the established parameters; and
- The parts are measured and the data transposed into the Destra software (Q-DAS GmbH, Weinheim, Germany), with the order of the measurements not being important.

D and H dimensions of the parts were measured using the Mitutoyo 500-196-30 digital scale caliper (Mitutoyo Corporation, Kawasaki, Japan). System and process capability is determined by calculating the capability coefficients described in Equation (1). The lower specification limit (LSL) and upper specification limit (USL) are the targets set for the process. The potential machine and process capability indices (C_m_, C_p_) represent the number of times the process spread fits into the tolerance interval. A high potential capability index does not guarantee that the process is close to the target value, which is why the position of the process spread in relation to the tolerance interval is determined by calculating the critical capability machine/process index (C_mk_/C_pk_).

(1) $$\left\{\begin{array}{c} C_{i}=\frac{USL-LSL}{x_{i\_99.865\%}-x_{i\_0.135\%}}\\ C_{ik}=min\left\{\frac{USL-x_{i_{50}\%}}{x_{i_{99.865}\%}-x_{i_{50}\%}},\frac{x_{i_{50}\%}-LSL}{x_{i_{50}\%}-x_{i_{0.135}\%}}\right\}\\ C_{target}=1.67\left(1.33\right) \end{array}\right.,i=\left\{m-machine,p-process\right\}$$

The location and dispersion were calculated using the M1_1,6_ method according to the ISO 22514-2: 2017 standard [21]. Subscripts 1 and 6 refer to equations for calculating the estimator for the location and dispersion, respectively. This means that the arithmetic mean of the values is used for the location being assumed, and externally tested the normal distribution, and the distance between the edges 0.135% and 99.865% for dispersion. The reference interval of the product characteristic is bounded by the 99.865% distribution quantile, and the 0.135% distribution quantile. The length of the interval is X_99,865%_−X_0,135%_ [35]. X_50%_ represents the 50% distribution quantile.

The results of the short-term and long-term capabilities were analyzed based on the requirements from the SPC Reference Manual, from Automotive Industry Action Group (AIAG) [19]. Destra software [49] was used to perform the capability study. The capability can be evaluated graphically by drawing capability histograms and capability plots. The requirements for indices C_m_, C_mk_, C_p_, and C_pk_ demand a minimum value of 1.67 for all of them. 

### 2.5. Capable Tolerance Specification for PolyJet Technology

Tolerance specification (tolerance, lower and upper limits) for the dimensions of the 3D printed circular part was chosen based on the general tolerances standards [34] and plastics molded parts tolerances [33]. A tolerance of ±0.1 mm was selected. Based on this specification, the AM system and process capability for PolyJet technology was calculated. The capability indices were compared with a capability target index of 1.67.

Rather than estimating the process capability for a particular tolerance, a capable tolerance and its limit deviations were calculated based on a target capability index. The target capability index was set to 1.67. The index of the process K was calculated using Equation (2) and describes the level by which the process is off target value and represents an appropriate measure of process centering [37,50]. The lower (LSL_T_) and upper (USL_T_) specification limits of the capable tolerance were calculated based on Equation (3). Upper limit deviation (ULD) and lower limit deviation (LLD) from nominal size were then determined based on Equation (3).

(2) $$K=\frac{\left(USL+LSL\right)-2x_{mean}}{USL-LSL}$$

The process mean is positioned between the midpoint of the specifications and one of the required limits if 0< |K|<1. |K|>1 indicates that the process mean is situated outside the required limits.

(3) $$\begin{array}{l} if\;K>0\;then\;\left\{\begin{array}{c} LSL_{T}=X_{50\%}-C_{Pk}\left(X_{50\%}-X_{0.135\%}\right)\\ LLD=T_{m}-LSL_{T}\\ USL_{T}=LSL+C_{P}\left(X_{99.865\%}-X_{0.135\%}\right)\\ ULD=USL_{T}-T_{m} \end{array}\right.\\ \begin{array}{c} if\;K<0\;then\;\left\{\begin{array}{c} USL_{T}=X_{50\%}+C_{Pk}\left(X_{99.865\%}-X_{50\%}\right)\\ ULD=USL_{T}-T_{m}\\ LSL_{T}=USL-C_{P}\left(X_{99.865\%}-X_{0.135\%}\right)\\ LLD=T_{m}-LSL_{T} \end{array}\right. \end{array} \end{array}$$

The capable lower limit deviation and capable upper limit deviation were determined based on the relations LLD_C_ < LLD_T_ and ULD_C_ >ULD_T_. The capable tolerance is calculated as follows: T_c_ = ULD_C_ – LLD_C_. A confirmatory analysis of AM process capability was performed using the determined capable tolerance.

## 3. Results and Discussion

### 3.1. The Variability of the Measurement System

The variance components (VarComp) compare the variation from each source of measurement error to the total variation. In these results, the %Contribution column (Table 2) shows that the variation from Part-To-Part for H and D dimension was 99.46%/99.12%, which is much larger than the total Gage R&R, which was 0.54%/0.88%. Thus, the largest part of the variation was due to the differences between parts. This means that the measurement system can reliably distinguish between parts. 

The measurement system variation compared to the total variation is shown in Table 3 and Table 4. The total Gage R&R equaled 7.33%/9.37% of the study variation for the H and D dimensions. In order to evaluate the capability of the measurement system to evaluate parts versus specification, the values %Tolerance are used, these values being calculated for each characteristic as the ratio between the study variation for each source and the process tolerance. 

The repeatability variation and the reproducibility variation, which shows the equipment variation (EV) and the appraiser variation (AV), respectively, were lower than 10%. Based on the requirements specified in the MSA 4 [46], the measurement system can be accepted. The number of distinct categories was greater than five (Table 3 and Table 4), resulting in an acceptable measurement system [46]. 

**Figure 4:**
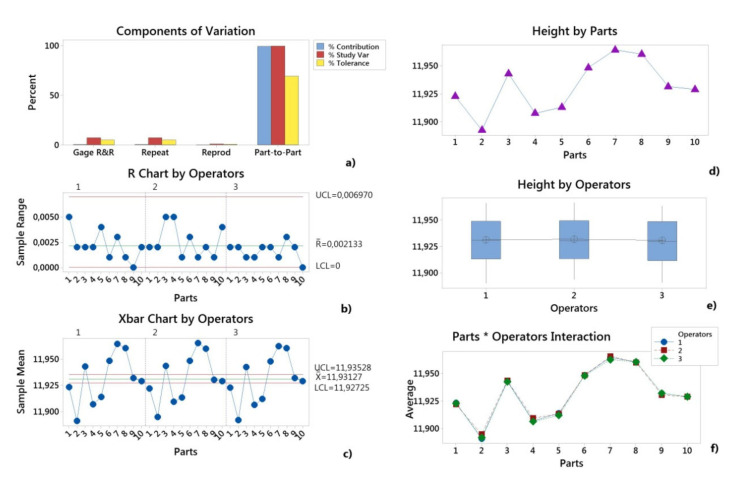
Gage R&R (ANOVA) report for height (H): (**a**) Components of Variation graph; (**b**) R Chart by Operator; (**c**) Xbar Chart by Operators; (**d**) By Parts graph; (**e**) By Operators graph; (**f**) Parts * Operators interaction.

**Figure 5:**
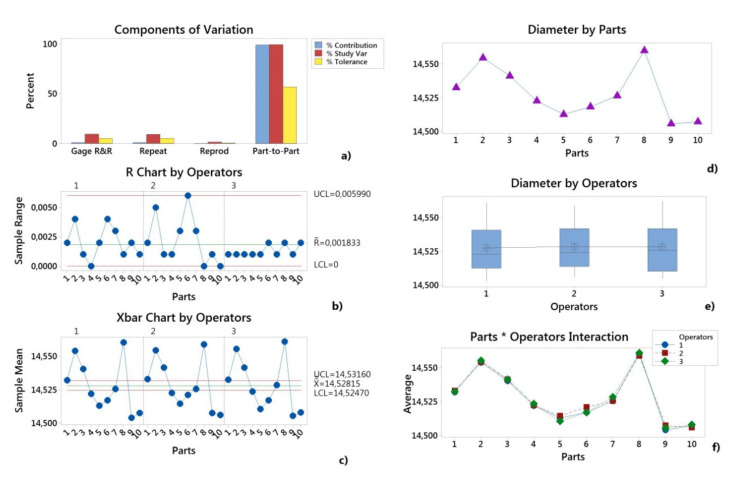
Gage R&R (ANOVA) report for diameter (D): (**a**) Components of Variation graph; (**b**) R Chart by Operator; (**c**) Xbar Chart by Operators; (**d**) By Parts graph; (**e**) By Operators graph; (**f**) Parts*Operators interaction.

The variability results of the measurement system are graphically provided in Figure 4 and Figure 5. In the Components of Variation graph, the %Contribution from Part-To-Part is larger than that of the total Gage R&R. Thus, much of the variation is due to differences between parts. The R Chart by Operator shows that Operators measured parts consistently. In the Xbar Chart by Operator, most of the points were outside the control limits. Thus, much of the variation is due to differences between parts.

The By Operator graphs (Figure 4e and Figure 5e) show that the differences between operators were smaller than the differences between parts. In the Parts * Operators Interaction graphs (Figure 4f and Figure 5f), the lines were approximately parallel and the p-value for the Parts * Operators interaction was 0.779/0.195 for the H and D dimensions. This indicates that no significant interaction between each Parts and Operators exists.

The Gage R&R result shows that for the height as well as for the diameter, a variation due to the measurement system was much lower than the part-to-part variation, as a result, the next studies could be based on measurements. 

### 3.2. System Performance of Objet EDEN 350 PolyJet

**Figure 6:**
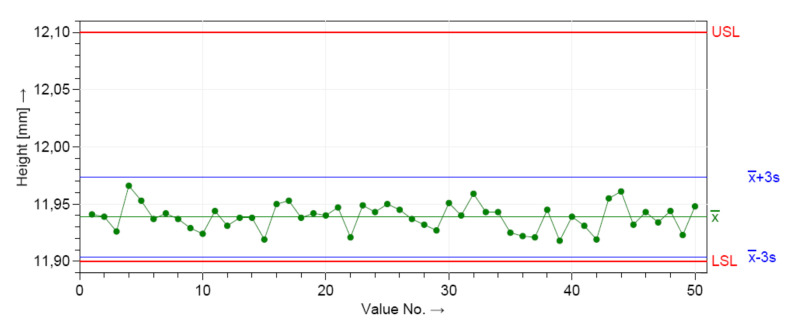
Control chart for the short-term capability study of the height dimension (H).

**Figure 7:**
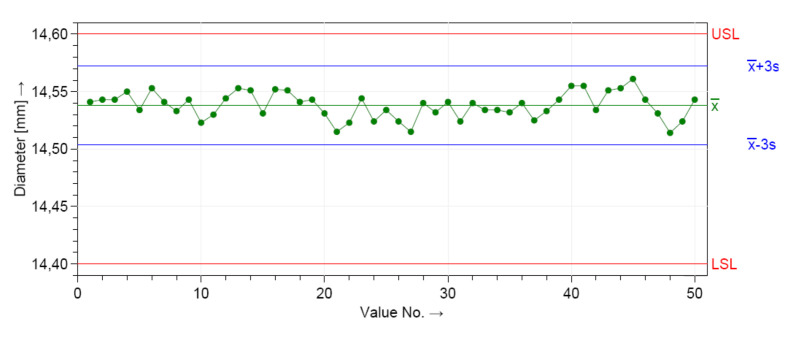
Control chart for the short-term capability study of the diameter dimension (D).

First, both critical assumptions for performing the machine capability (system performance) analyses were graphically checked. The control charts from Figure 6 and Figure 7 show the manufacturing process information for all 50 measurements of the D and H measured dimensions. The distributions were stable over the period of study, as shown in Figure 6 and Figure 7.

**Figure 8:**
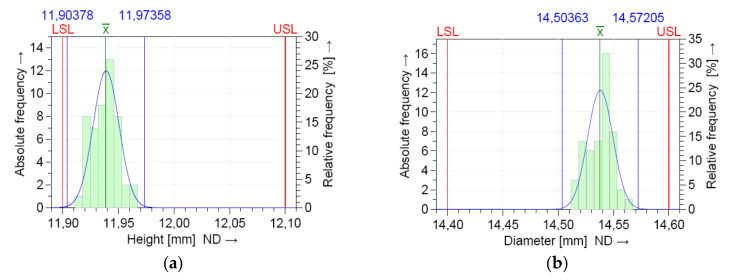
Histogram of the individuals and the distribution model for short term capability study: (**a**) height (H); (**b**) diameter (D).

The dimensional values lay within the LSL and USL, indicating that the process is in statistical control for both dimensions. A normal distribution was detected based on the Anderson–Darling normality test. Figure 8 shows the histograms of the individuals and the distribution models. 

The measurements were located near the upper specification limit (USL) of the diameter (D) and near the lower specification limits (LSL) of the height (H), respectively. This graphics show the shape of the subgroup frequencies.

**Figure 9:**
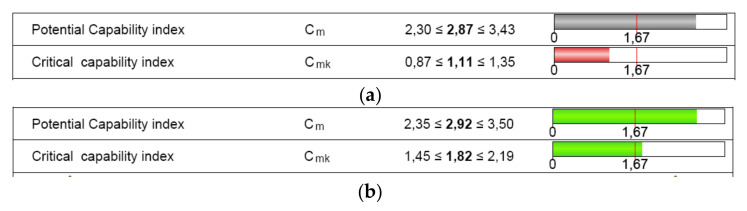
Machine capability analysis report for: (**a**) dimension H; (**b**) dimension D.

The numerical results of the machine capability analysis are shown in Table 5 and Table 6 for both dimensions of the circular specimen, where Tm is the tolerance center, T is the tolerance of the characteristic, n is the sample size, *x_min_* the minimum value of the characteristic, *x_max_* is the maximum value of the characteristic, *x*_mean_ is the median of all values, StDev is the standard deviation of all individuals, X_0.135%_ is the 0.135% distribution quantile, X_50%_ is the 50% distribution quantile, and X_99.865%_ is the 99.865% distribution quantile. The potential and the critical capability index both showed three values (Figure 9) that specify the two-sided 95% confidence interval for the respective capability index: lower confidence limit, estimator, and upper confidence limit.

The requirements for indices C_m_ and C_mk_ were met for the D dimension (Figure 9b). Based on the measured parts, the critical capability index was lower than the target for the characteristic H. Therefore, the 3D printer capability was not proven (Figure 9a). 

### 3.3. Process Capability of PolyJet

**Figure 10:**
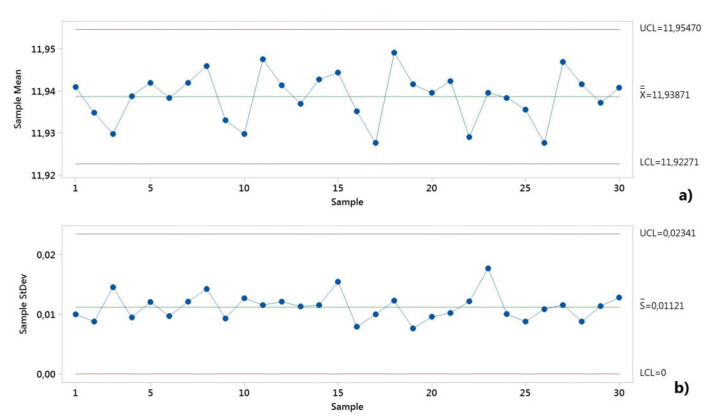
The control charts for the long-term capability study of height dimension (H): (**a**) mean data; (**b**) standard deviation data.

**Figure 11:**
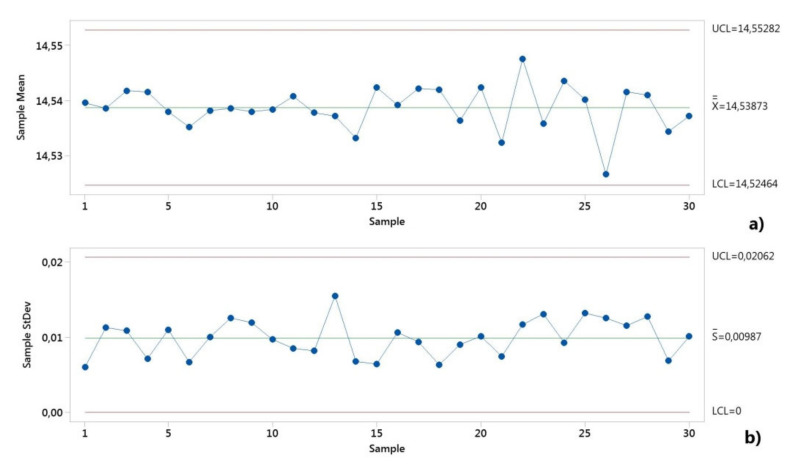
The control charts for the long-term capability study of diameter dimension (D): (**a**) mean data; (**b**) standard deviation data.

The control charts of the process capability for both dimensions of the diameter and height of the circular specimen are shown in Figure 10 and Figure 11. The Xbar-S control charts for the subgroups with the sample size of five pieces were chosen to check if the process variation was in control. The mean data and standard deviation data showed that none of the points were outside the control limits (UCL, upper control limit, LCL, lower control limit), and the points displayed a random pattern. Thus, the process variation was in control. 

**Figure 12:**
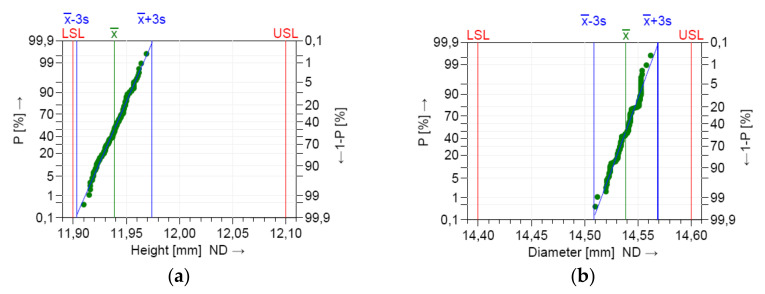
Normal probability plot graph for the long-term capability study: (**a**) height dimension (H); (**b**) diameter dimension (D).

The model distribution of the data for the dimensions H and D showed a normal distribution, as shown in Figure 12. The entire production process was stable and controllable.

**Figure 13:**
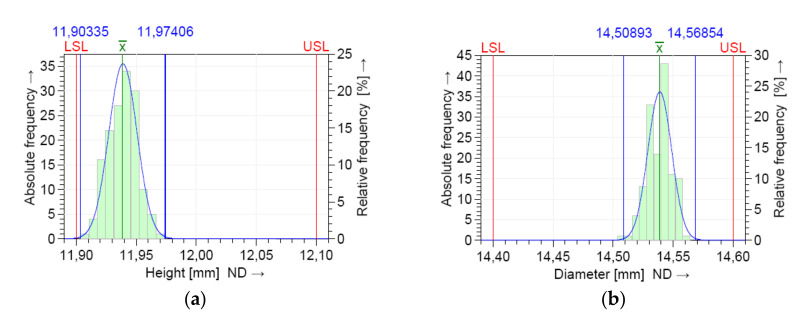
Histogram of individuals and the distribution model for the long-term capability study: (**a**) height dimension (H); (**b**) diameter dimension (D).

The location of the process distribution (Figure 13) was near the upper tolerance limits for the dimension of diameter (D) and near the lower tolerance limits for the dimension of height (H), respectively.

The numerical results of the process capability analysis are shown in Table 7 and Table 8 for both dimensions of diameter and height. The standard deviation of height was slightly larger than that of diameter.

**Figure 14:**
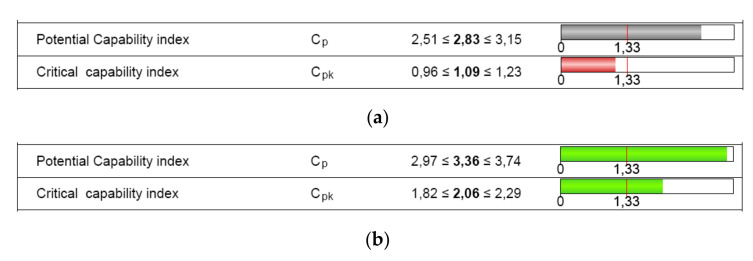
Process capability analysis report: (**a**) height dimension (H); (**b**) diameter dimension (D).

Based on the requirements, the target for C_pk_ is very often established at a minimum of 1.67. Some of the industry manufacturers accept even lower values of 1.33 for C_p_ and C_pk_ [51]. Even so, the result for the height characteristic in terms of C_pk_ was lower than 1.67 or 1.33. The requirements for indices C_pm_ and C_pk_ were met for the diameter dimension (Figure 14), but were not met for the height dimension.

### 3.4. Capable Tolerance and Its Limits Deviation for PolyJet Process

The calculation of the capability indices was based on the location and dispersion of the characteristic value with respect to the specified tolerance. x_mean_ indicates the location of the process. It can be observed from the process capability graphics (Figure 13) that the x_mean_ was lower than the nominal value for the H characteristic and higher for the D characteristic, respectively.

Capable tolerance and its limit deviations were calculated based on a target capability index of 1.67 for both dimensions of height and diameter. The index of process K was calculated, and the results showed the value of 0.62 for the height and −0.38 for the diameter, respectively. The capable lower limit deviation and capable upper limit deviation were determined for both dimensions.

The capable limit deviations of the circular specimen were found as ULD = max{ULD_D_, ULD_H_} and LLD = min{LLD_D_, LLD_H_}, where the subscripts H and D represent the characteristic height and diameter, respectively. The results show that the capable lower limit deviation and capable upper limit deviation of the circular artifact were LLD = −0.13 mm and ULD = +0.09 mm, respectively. The capable tolerance interval of the circular artifact was T_C_ = 0.22 mm.

**Figure 15:**
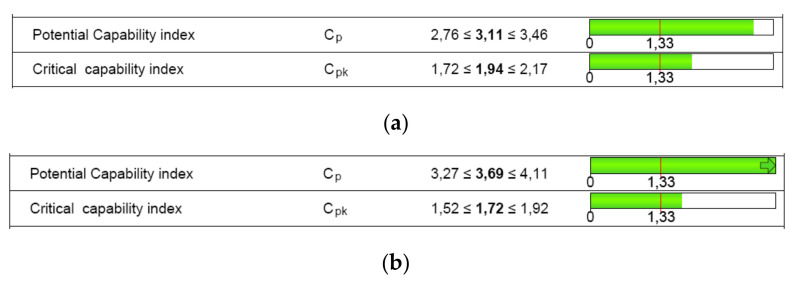
Process capability analysis report based on the capable tolerance of the circular artifact: (**a**) height dimension (H); (**b**) diameter dimension (D).

A confirmatory analysis of AM process capability was performed using the determined capable tolerance of the circular artifact. The process capability result was “too high” (C_pk_>1.67), as shown in Figure 15. Thus, the requirements were met.

### 3.5. Determination of Tolerance Grade (ISO IT grade)

Tolerance grades indicate the degree of accuracy of manufacture. Since IT grades provide guidance on how precise a manufactured feature of a particular size should be, they can be used to compare different manufacturing processes [52]. The lower value of IT Grade implies a better dimensional accuracy. The IT Grade was calculated for 50 specimens of the circular artifact, based on the standard ISO 286 specifications [32]. The dimensional accuracy and IT Grade depend on the size of the feature. Two dimensions of the circular artifact, the height and the diameter were analyzed. These sizes were within the ISO basic size range of (10–18 mm).

(4) $$n_{i}=\frac{\left|D_{N}-D_{M}{}_{i}\right|}{0.45{\left(\sqrt{D_{min}D_{max}}\right)}^{\frac{1}{3}}+0.001\sqrt{D_{min}D_{max}}},i=\left\{1,\ldots ,50\right\}$$

The relative magnitude of each IT (International Tolerance) Grade is calculated relative to the standard tolerance unit i. The standard tolerance unit is i = 1.083 μm for the ISO basic size range of D_min_ = 10 mm to D_max_ = 18 mm. The tolerance unit “n” was calculated using Equation (4), where ‘D’ is the geometric mean of the ISO basic size range; D_N_ is the nominal dimension; and D_M_ is the measured dimension. Table 9 shows the IT grades for the height (H), and the diameter (D) of the circular specimen. The tolerance grades were determined based on the tolerance unit n.

**Figure 16:**
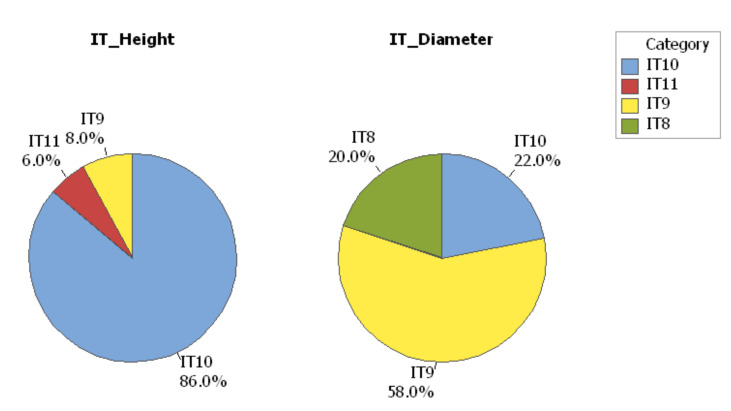
IT grades for the characteristics of height and diameter of the circular artifact within the size range (10–18 mm).

The results show that the International Tolerance Grade of the height dimension was IT10 for 86% of specimens (Figure 16). A significant variation in the IT Grade percent was detected for the diameter dimension with a 58% IT10 distribution, as shown in Figure 16. The IT Grade, which represents the dimensional accuracy of the AM systems for each interval of ISO basic sizes can be determined using the same procedure.

### 3.6. Quality Inspection through Microscopy Analysis

**Figure 17:**
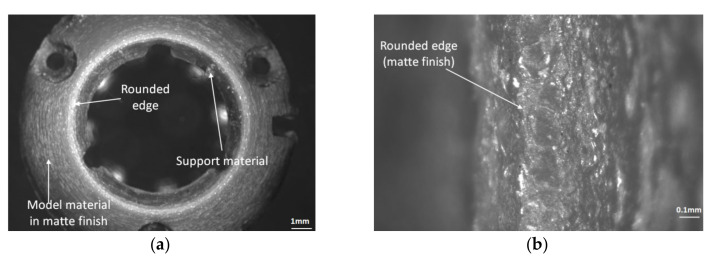
The lower surface of the circular artifact manufactured by the Objet EDEN 350 PolyJet. (**a**) Lower surface detail affected by support material; (**b**) lower edge detail in a matte finish.

**Figure 18:**
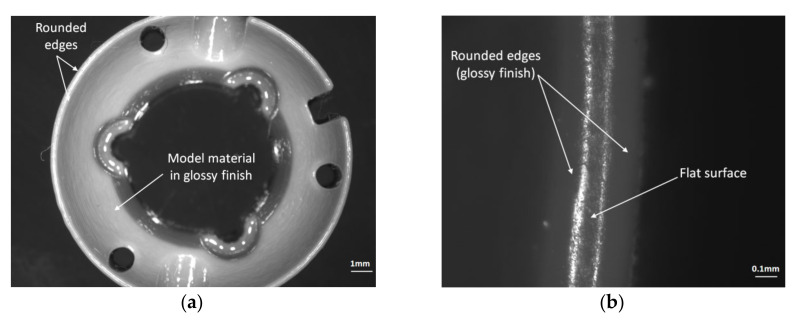
The upper surface of the circular artifact manufactured by the Objet EDEN 350 PolyJet. (**a**) Upper surface in glossy mode; (**b**) upper edge detail in a glossy finish.

A microscopy analysis study was performed to conduct a quality inspection of the critical features of the circular workpiece. The dimension H was measured between the upper and lower surface of the specimen, and dimension D on the upper surface of the workpiece, respectively. The quality of these surfaces should be investigated. There was no support material deposited on the upper surfaces of the model printed in glossy mode, only on the bottom surfaces, as shown in Figure 17 and Figure 18. 

The lower surface of the circular specimen was affected by the material support. Small pieces of support material were detected on the lower surface of the specimen, even if the specimen was cleaned with a pressure water jet after 3D printing (Figure 17). 

The quality of the lower and upper edges of the circular artifact may influence the artifact height dimension. A good quality surface without material defects was detected on the upper edge of the specimen (Figure 18b). The edges of the upper surface printed in glossy mode were rounded, as shown in Figure 18b. Additionally, the sharp edge of the lower surface affected by the support material was rounded, as shown in Figure 17. This edge roundness can explain why the distribution of the height measurements was located near the lower tolerance limits and was lower than the nominal value.

**Figure 19:**
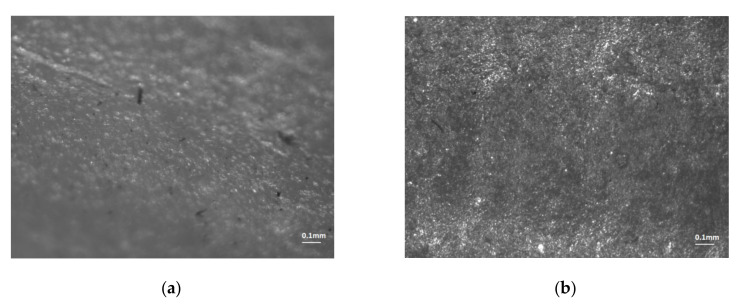
Microscopic views (1:1 × 10^−4^ m scale) of the lateral surface of the artifact in the glossy finish area located: (**a**) parallel to the X-axis (0°); (**b**) parallel to the Y-axis (90°).

**Figure 20:**
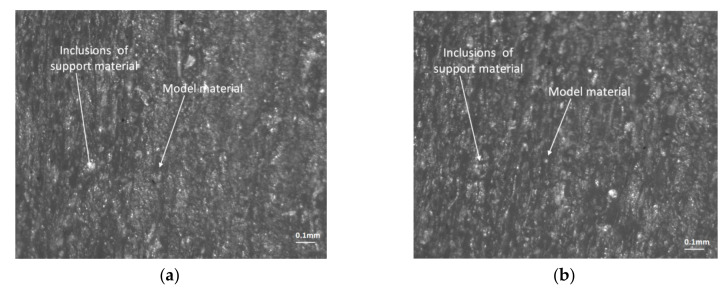
Microscopic views (1:1 × 10^−4^ m scale) on the lateral surface of the artifact, in the matte finish area affected by the support material located: (**a**) parallel to the X-axis (0°); (**b**) parallel to the Y-axis (90°).

For both the glossy and matte finishes, microscopic investigations on the lateral surface of the circular artifact were conducted on a perpendicular and parallel direction to the X-axis (Figure 19 and Figure 20). It can be seen as a clean and smooth surface in the X-axis direction (Figure 19a) for a glossy finish. Rough areas were detected on the perpendicular direction to the X-axis (Figure 19b). The steep surface affected by the support material indicates a homogenous material that contained small inclusions of the FullCure 705 support material (Figure 20).

## 4. Conclusions

This paper contributes to the characterization of the dimensional accuracy, repeatability, system performance, and process capability of polymer-based AM processes and systems. The methodology used for quality control in additive manufacturing allows the polymer-based AM processes to be implemented in production. Additionally, this methodology can be used as the AM’s machine monitoring technique.

The following conclusions are drawn:

- The properties of the polymers used in additive manufacturing processes are relevant to the dimensional accuracy of the parts and require different evaluation and quantification of geometrical tolerances in comparison to metal materials and other plastics.
- The implementation of AM for pre-production series and short series production mainly depends on the repeatability, machine capability, and process capability.
- The values of the system and process capability indices (C_m_, C_mk_, C_p_, and C_pk_) of the circular parts produced with Objet VeroBlue RGD840 material by PolyJet technology were greater than 1.67 within the capable tolerance interval of 0.22 mm. The capable lower limit deviation and capable upper limit deviation of the circular artifact were −0.13 mm and +0.09 mm, respectively.
- From the statistical analysis conducted on the geometrical dimensions of the circular parts, the distribution of the measurements showed that they were not centered on the nominal value. These were located near the upper tolerance limits for the dimension of diameter (D) and near the lower tolerance limits for the dimension of height (H), respectively.
- The roundness of the artifact edges detected through the microscopy investigations explains why the distribution of the height measurements was located near the lower tolerance limits and was lower than the nominal value. Additionally, the height values resulting from the measurements were lower than the nominal value.
- The International Tolerance Grade for polymer manufactured circular parts was found to be between IT8 to IT10, which is in-line as per the ISO-286 for materials. The IT Grade of the height dimension was IT10 for 86% of specimens and 58% for the diameter dimension, respectively.
- A small size specimen, built in a minimum of 50 pieces, should be used for AM system capability determination to minimize material consumption and related costs. Three batches of 50 specimens should be built for the process capability study.

Further research is required for the capability characterization of other AM machines and processes using different types of materials.

## Figures and Tables

**Table 1:** Objet VeroBlue RGD840 properties [39].

Property	ASTM	Metric
Tensile Strength	D-638-03	50–60 MPa
Elongation at Break	D-638-05	15%–25%
Flexural Strength	D-790-03	60–70 MPa
Rockwell Hardness	Scale M	73–76 Scale M
Water Absorption	D-570-98 24hr	1.5%–2.2%

**Table 2:** Variance components for the characteristic diameter and height.

Source	VarComp ^1^	Contribution ^1^	VarComp ^2^	Contribution ^2^
Total Gage R&R	0.0000032	0.88%	0.0000029	0.54%
Repeatability	0.0000031	0.86%	0.0000028	0.52%
Reproducibility	0.0000001	0.02%	0.0000001	0.01%
Operators	0.0000001	0.02%	0.0000001	0.01%
Part-To-Part	0.0003585	99.12%	0.0005336	99.46%
Total Variation	0.0003617	100%	0.0005365	100%

^1^ Diameter, ^2^ Height.

**Table 3:** Gage evaluation for diameter (D).

		Study Var	%Study Var	%Tolerance
Source	StdDev (SD)	(6 × SD)	(SV)	(SV/Toler)
Total Gage R&R	0.0017829	0.010698	9.37%	5.35
Repeatability	0.0017611	0.010566	9.26%	5.28
Reproducibility	0.0002783	0.00167	1.46%	0.83
Operators	0.0002783	0.00167	1.46%	0.83
Part-To-Part	0.0189344	0.113606	99.56%	56.8
Total Variation	0.0190181	0.114109	100%	57.05
Number of Distinct Categories = 14				

**Table 4:** Gage evaluation for height (H).

		Study Var	%Study Var	%Tolerance
Source	StdDev (SD)	(6 × SD)	(SV)	(SV/Toler)
Total Gage R&R	0.0016968	0.010181	7.33%	5.09
Repeatability	0.0016783	0.01007	7.25%	5.03
Reproducibility	0.00025	0.0015	1.08%	0.75
Operators	0.00025	0.0015	1.08%	0.75
Part-To-Part	0.0231008	0.138605	99.73%	69.3
Total Variation	0.0231631	0.138978	100%	69.49
Number of Distinct Categories = 16				

**Table 5:** Machine capability analysis for the H dimension.

Drawing Values		Collected Values		Statistics	
Tm	12	n	50	StDev	0.0116
LSL	11.9	x_min_	11.918	X_0.135%_	11.90378
USL	12.1	x_max_	11.966	X_99.865%_	11.97358
T	0.2	x_mean_	11.939	X_50%_	11.93868

**Table 6:** Machine capability analysis for the D dimension.

Drawing Values		Collected Values		Statistics	
Tm	14.5	n	50	StDev	0.0114
LSL	14.4	x_min_	14.514	X_0.135%_	14.50363
USL	14.6	x_max_	11.561	X_99.865%_	14.57205
T	0.2	x_mean_	14.54	X_50%_	14.53784

**Table 7:** Process capability analysis for the H dimension.

Drawing Values		Collected Values		Statistics	
Tm	12	n	150	StDev	0.0118
LSL	11.9	x_min_	11.910	X_0.135%_	11.90335
USL	12.1	x_max_	11.969	X_99.865%_	11.97406
T	0.2	x_mean_	11.939	X_50%_	11.93871

**Table 8:** Process capability analysis for the D dimension.

Drawing Values		Collected Values		Statistics	
Tm	14.5	n	150	StDev	0.00994
LSL	14.4	x_min_	14.510	X_0.135%_	14.50893
USL	14.6	x_max_	14.562	X_99.865%_	14.56854
T	0.2	x_mean_	14.540	X_50%_	14.53873

**Table 9:** International tolerance grades for the circular artifact.

ISO 286 Standard Requirements		IT8	IT9	IT10	IT11
Max magnitude of the tolerance zone		25 i	40 i	64 i	100 i
Size range (10–18 mm), i = 1.083 μm		27 μm	43 μm	70 μm	109 μm
**Collected values**					
n	Linear dimension (Height = 12 mm)	-	(32–42) μm	(44–69) μm	(73–75) μm
	Radial dimension (Diameter = 14.5 mm)	(19–24) μm	(28–41) μm	(47–57) μm	-
